# *M*_4_Au_12_Ag_32_(*p*-MBA)_30_ (*M* = Na, Cs) bimetallic monolayer-protected clusters: synthesis and structure

**DOI:** 10.1107/S2056989018008393

**Published:** 2018-06-19

**Authors:** Brian E. Conn, Badri Bhattarai, Aydar Atnagulov, Bokwon Yoon, Uzi Landman, Terry P. Bigioni

**Affiliations:** a Department of Chemistry, University of Toledo, Toledo, Ohio 43606, USA; b School of Physics, Georgia Institute of Technology, Atlanta, Georgia 30332 0430, USA

**Keywords:** crystal structure, silver nanoparticle, gold nanoparticle, bimetallic nanoparticle, monolayer-protected cluster, Bader analysis

## Abstract

Crystals of *M*_4_Au_12_Ag_32_(*p*-MBA)_30_ bimetallic monolayer-protected clusters (MPCs), where *p*-MBA is *p*-mercapto­benzoic acid and *M* ^+^ is a counter-cation (*M* = Na, Cs) have been grown and their structure determined. The mol­ecular structure of triacontakis[(4-carboxylatophenyl)sulfanido]dodecagolddotriacontasilver, Au_12_Ag_32_(C_7_H_5_O_2_S)_30_ or C_210_H_150_Ag_32_Au_12_O_60_S_30_, exhib­its point group symmetry  at 100 K. The overall diameter of the MPC is approximately 28 Å, while the diameter of the Au_12_Ag_20_ metallic core is 9 Å. The structure displays ligand bundling and inter­molecular hydrogen bonding, which gives rise to a framework structure with 52% solvent-filled void space. The positions of the *M* ^+^ cations and the DMF solvent mol­ecules within the void space of the crystal could not be determined. Three out of the five crystallographically independent ligands in the asymmetric unit cell are disordered over two sets of sites. Comparisons are made to the all-silver *M*_4_Ag_44_(*p*-MBA)_30_ MPCs and to expectations based on density functional theory.

## Abstract 2

The synthesis and structure of mixed gold/silver *M*_4_Au_12_Ag_32_(*p*-MBA)_30_ bimetallic monolayer-protected clusters is reported and compared to that of silver *M*_4_Ag_44_(*p*-MBA)_30_ monolayer-protected clusters (*M* = Na, Cs).

## Chemical context   

The *M*_4_Ag_44_(*p*-MBA)_30_ monolayer-protected cluster (MPC) has been studied in detail previously, where *M* ^+^ is an alkali metal counter-ion (*M* = Na, Cs) and *p*-MBA is *p*-mercapto­benzoic acid (Desireddy *et al.*, 2013 ▸; Conn *et al.*, 2015 ▸), along with other related 44 silver-atom species (Bakr *et al.*, 2009 ▸; Pelton *et al.*, 2012 ▸; AbdulHalim *et al.*, 2013 ▸; Yang *et al.*, 2013 ▸; Chakraborty *et al.*, 2013 ▸). The formula has been shown to be Na_4_Ag_44_(*p*-MBA)_30_ and K_4_Ag_44_(*p*-MBA)_30_ in all-sodium and all-potassium preparations, respectively, and the mol­ecular and crystal structures have been determined crystallo­graphically (Desireddy *et al.*, 2013 ▸). The crystal was determined to have a framework structure, with 52% solvent-filled void space, that is a consequence of both ligand bundling and inter­particle hydrogen bonding (Yoon *et al.*, 2014 ▸). The positions of the alkali metal counter-ions were not determined, and are presumably located in the solvent portion of the crystal (Desireddy *et al.*, 2013 ▸).

Structurally related species have also been prepared with non-polar ligands, using non-polar synthetic conditions, forming chemically distinct members of the 44 silver-atom family of species, *e.g*. (PPh_4_)_4_Ag_44_(SPhF_2_)_30_ along with SPhF and SPhCF_3_ variants (Bakr *et al.*, 2009 ▸; Yang *et al.*, 2013 ▸). In the crystals of these species, the ligand bundling is not dominant and ligand inter­actions do not lead to framework structures. Instead, ligands pack more tightly, with 36% solvent-filled void space, and the bulky PPh_4_^+^ counter-cations lock into place in the crystals such that they can be located (Yang *et al.*, 2013 ▸).

Silver and gold mix readily to form the naturally occurring alloy electrum and therefore the study of mixtures of silver and gold within these MPCs is of inter­est (Yang *et al.*, 2013 ▸). Mixtures of *M*_4_Au_*x*_Ag_44–*x*_(*p*-MBA)_30_ MPCs can be obtained by co-reducing silver and gold polymers of *p*-MBA, where 0 ≤ *x* ≤ 12 (Conn *et al.*, 2018 ▸). Gold-rich species have been synthesized and then thermally processed to destroy the species that contained fewer gold atoms, thereby enriching the samples in *M*_4_Au_12_Ag_32_(*p*-MBA)_30_ MPCs.

Once high-purity samples of *M*_4_Au_12_Ag_32_(*p*-MBA)_30_ MPCs had been prepared and crystallized, the locations of the gold atoms could be determined by crystallographic methods. Prior reports using non-polar members of the 44 metal-atom family of species determined that the 12 gold atoms are located in the icosa­hedral inner core of that mol­ecule (Yang *et al.*, 2013 ▸). It is not clear, however, whether different synthetic conditions, ligands, and solvent class would affect the synthetic mechanism, electronic structure, and ultimately the organ­iza­tion of metal atoms within the core. We present here the chemical synthetic method of producing *M*_4_Au_12_Ag_32_(*p*-MBA)_30_ MPCs as well as their X-ray determined structure and verify that the gold atoms are indeed located in the core of this MPC. Comparisons with other family members are also made to examine the effects of heteroligands and heteroatoms on the structures of these species.
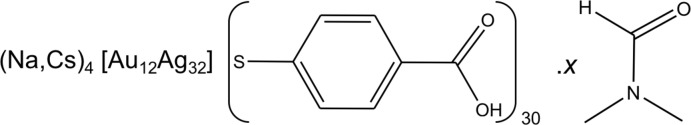


## Structural commentary   

There are four sets of chemically equivalent positions for metal atoms in the Au_12_Ag_32_(*p*-MBA)_30_^4−^ mol­ecular structure. All 12 positions in the icosa­hedral inner core are chemically equivalent, whereas the dodeca­hedral outer core contains a set of eight chemically equivalent positions (defining a cube) and a set of 12 chemically equivalent positions (a pair of atoms beneath each of the six mounts). The remaining 12 metal atoms are found in pairs in the six mounts and are chemically equivalent. In principle, then, there are three possible ways to locate 12 equivalent gold heteroatoms.

Density-functional calculations (Kresse & Joubert, 1999 ▸; Perdew, 1991 ▸; Perdew *et al.*, 1992 ▸, 1993 ▸) were performed to evaluate the energy differences upon substitution of gold atoms into each of these four distinct metal-atom positions. Each calculation was done for a *M*_4_AuAg_43_(*p*-MBA)_30_ MPC, the structures of which were relaxed after substitution. In each case, the energy of *M*_4_AuAg_43_(*p*-MBA)_30_ was found to be lower than *M*_4_Ag_44_(*p*-MBA)_30_. It was found that substitution of gold atoms into the icosa­hedral core has the biggest effect, lowering the energy by 0.71 eV per Au atom. The next most energetically favorable position was that of the eight atoms in the dodeca­hedral shell, lowering the energy by 0.30 eV per Au atom; these positions are of particular inter­est since they are the only atoms in the metal core that are exposed and capable of directly inter­acting and reacting with other species in solution. The least favorable positions for substitution were found to be the pairs of metal atoms in the mounts, lowering the energy by 0.170 eV per Au atom, and the pairs of metal atoms beneath the mounts, lowering the energy by 0.13 eV per Au atom. Based on these calculations, the 12 substituted gold atoms were expected to be found in the icosa­hedral core.

**Figure 1:**
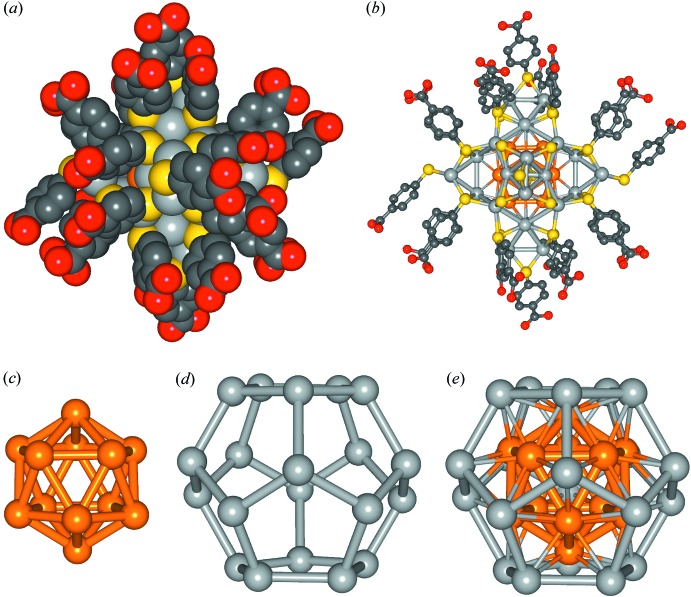
Structure of Au_12_Ag_32_(*p*-MBA)_30_^4−^. Complete X-ray-determined structure shown in (*a*) space-filling view and (*b*) ball-and-stick view (out-of-plane ligands removed for clarity). The core structure is shown as (*c*) an Au_12_ icosa­hedral inner shell, which is nested inside of (*d*) an Ag_20_ dodeca­hedral outer shell, together making (*e*) a bimetallic 32-atom excavated dodeca­hedral core. Other colors: red – O; grey – C; yellow – S (H not shown). The overall diameter of the MPC was measured to be about 28 Å, while the diameter of the inorganic portion of the structure was 17 Å and the metallic Au_12_Ag_20_ dodeca­hedral core was 9 Å. Measurements were made between the centers of opposing atoms in the structure.

**Figure 2:**
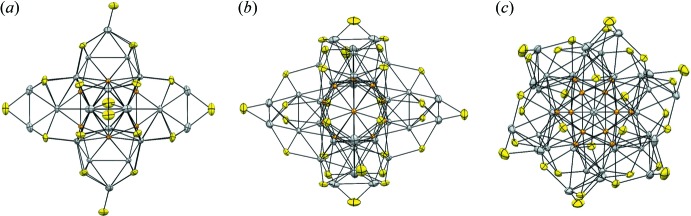
Structure of Au_12_Ag_32_(*p*-MBA)_30_^4−^ using displacement ellipsoids that were drawn at the 50% probability level for three different views of the structure. Au atoms are depicted in orange, Ag atoms in grey, and S atoms in yellow. Views are (*a*) down a fourfold axis of the pseudo­octa­hedral structure, (*b*) with one 31.7° rotation from (*a*) about the horizontal axis, and (*c*) with two 45° rotations from (*a*) about the horizontal and vertical axes. The organic portion of the mol­ecule was omitted for clarity.

The positions of the 12 Au atoms were determined by single-crystal X-ray crystallographic methods. The full refinement of the Au_12_Ag_32_(*p*-MBA)_30_^4−^ mol­ecular structure revealed that the 12 gold atoms reside in the icosa­hedral inner core of the MPC. The structure consists of a 12 gold-atom icosa­hedron surrounded by a 20 silver-atom dodeca­hedron, forming a 32-atom excavated-dodeca­hedral bimetallic core. The metal core is capped by six equivalent Ag_2_(*p*-MBA)_5_ mount motifs, which are octa­hedrally located about the core (Fig. 1 ▸). The Au_12_Ag_32_(*p*-MBA)_30_^4−^ anion is located about an inversion center and exhibits point group symmetry 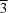 (Fig. 2 ▸).

The crystallographically determined locations of the 12 gold atoms in the icosa­hedral inner core of the bimetallic MPC are consistent with the expected locations based on our DFT calculations and based on previous reports (Yang *et al.*, 2013 ▸). In addition, this result is in agreement with the known properties of gold and silver. Although gold and silver are isoelectronic and have almost identical atomic radii, their chemical properties and bonding can be quite different. For example, Au—S and Ag—S bonding is typically two- and three-coordinate, respectively (Dance, 1986 ▸; Dance *et al.*, 1991 ▸), which makes the bonding of the gold heteroatoms incompatible with the structure of the protecting mounts (Desireddy *et al.*, 2013 ▸; Conn *et al.*, 2016 ▸). It is therefore unlikely that gold atoms would substitute into the ligand shell without changing the metal-atom count (Yang *et al.*, 2014 ▸).

Furthermore, gold is known to be more electronegative and more noble than silver, so the gold atoms are expected to assume positions within the structure where they can possess the lowest oxidation state among the metal atoms. Bader analysis (Bader, 1990 ▸; Tang *et al.*, 2009 ▸) of the electron distribution in *M*_4_Ag_44_(*p*-MBA)_30_ has shown that atoms in the inner icosa­hedral core have an oxidation state of zero (Conn *et al.*, 2015 ▸; Yang *et al.*, 2013 ▸) whereas in *M*_4_Au_12_Ag_32_(*p*-MBA)_30_ those atoms are slightly reduced (Conn *et al.*, 2018 ▸). The other metal atoms were found to be oxidized, with their oxidation states increasing with distance from the center of the mol­ecule. The X-ray-determined locations of the gold atoms in the inner core are therefore also consistent with the Bader analysis and the known properties of gold and silver.

The crystal structures of Ag_44_(*p*-MBA)_30_^4−^, Au_12_Ag_32_(*p*-MBA)_30_^4−^, Ag_44_(SPhF_2_)_30_^4−^ and Au_12_Ag_32_(SPhF_2_)_30_^4−^ were analyzed carefully to identify changes in the structure as a result of substituting 12 silver atoms for gold atoms. The metal–metal bond lengths within the 12-atom icosa­hedron, the 20-atom dodeca­hedron, and the mounts were compared for the two structures. The results of the bond-length analysis are reported in Table 1 ▸.

The Au—Au and Ag—Ag bonds in the bulk metals have similar bond lengths (2.884 and 2.889 Å, respectively; JCPDS no. 04-0784 and no. 04-0783, respectively; ICDD, 2015 ▸), and therefore substituting the two metals might not be expected to change bond lengths within the structures. This is not the case, however. The bond lengths within the 12-atom icosa­hedron were found to shorten from 2.825 ± 0.012 Å to 2.795 ± 0.013 Å when gold was incorporated, indicating stronger than expected bonding within the inner core. Bond lengths within the 20-atom dodeca­hedron were found to be essentially unchanged (3.175 ± 0.040 Å *versus* 3.190 ± 0.040 Å), however. The metal—metal bonds in the mounts were also found to be unaffected by the gold-atom substitution.

These changes in bond lengths may be the result of a change in the electron-density distribution due to the electrophilicity of the gold atoms in the inner icosa­hedral core, which tend to pull electron density from the outer dodeca­hedral core. For example, Bader analysis of the charge distribution shows that the number of excess electrons on the icosa­hedral core increases from 0.010 to 1.769 upon substitution of gold atoms. This reduction of the inner core is accompanied by a further oxidation of the silver atoms in the outer core, where the number of excess electrons decreases from −4.928 to −6.546 upon substitution of gold atoms. The gold-atom substitution into the core does not affect the charge density on the silver atoms in the mounts. The results of the Bader charge analysis are reported in Table 2 ▸.

Based on the Bader analysis, the redistribution of the electron density was found to be almost entirely confined to the 32-atom metal core (comprising the icosa­hedral and dodeca­hedral shells). While this appears to be the origin of the changes in metal—metal bond lengths inside the 32-atom metal core, it may also be the reason that the rest of the mol­ecule remains essentially unchanged by this metal-atom modification to the structure.

It is also inter­esting to note that classical electrostatics predicts that any charges carried by a metal sphere would be located on the surface of that sphere. The Bader charge analysis for *M*_4_Ag_44_(*p*-MBA)_30_ is in agreement with this classical picture, but that is not the case for *M*_4_Au_12_Ag_32_(*p*-MBA)_30_. In the former case, the inner core is neutral and all of the charge is located on the outer core. In the latter case, both the inner and outer core carry charge (in fact, the 32-atom metal core is polarized). This demonstrates the failure of the classical theory with regard to predicting charge distributions on such a small scale, because of finite screening lengths in real materials.

## Supra­molecular features   

Like the silver-only *M*_4_Ag_44_(*p*-MBA)_30_ MPCs, *M*_4_Au_12_Ag_32_(*p*-MBA)_30_ MPCs crystallize as framework structures as a consequence of intra­molecular ligand bundling and inter­molecular hydrogen bonding. The ligand bundling is a consequence of inter­actions between the ligands, with the magnitude of the inter-ligand van der Waals interaction energy calculated to be −0.95 eV/mount. The ligands form six dimer bundles, which are evenly spaced in the same plane, and six trimer bundles, with three above and three below the plane defined by the dimers. Together, the twelve bundles define the connectivity of the crystal’s framework structure such that the MPCs have pseudo-face-centered-cubic packing. The nature of the framework structure and hydrogen bonding in these materials was studied in detail in a previous report (Yoon *et al.*, 2014 ▸).

## Database survey   

It is instructive to compare the structures of the related but chemically distinct Au_12_Ag_32_(*p*-MBA)_30_^4−^ and Au_12_Ag_32_(SPhF_2_)_30_^4−^ species to examine the effect of ligand structure on crystal structure as well as the question of whether the composition of the outside of the MPC can affect the structure of the core. Likewise, the Ag_44_(*p*-MBA)_30_^4−^ and Au_12_Ag_32_(*p*-MBA)_30_^4−^ structures can be compared to address the question of whether the composition of the core can affect the ligand shell and crystal structure.

First, the crystal structures of the two species are entirely different, due to the different mechanisms of inter­actions between the MPCs. In the case of *p*-MBA, hydrogen bonding governs the inter­actions between the MPCs while ligand bundling within the ligand shell defines the directionality of those inter­actions (Yoon *et al.*, 2014 ▸). As a result, the overall structure of the crystal is that of a framework material with large void spaces (Yoon *et al.*, 2014 ▸). No such inter­actions exist in the crystals of hydro­phobic MPCs, and therefore the crystal structure is more compact with less well-defined inter­molecular inter­actions (Yang *et al.*, 2013 ▸). The difference in crystal structures due to the different ligands is also expected to lead to entirely different mechanical properties of these two crystalline materials (Yoon *et al.*, 2014 ▸). The observed differences in crystal structures are similar when comparing Ag_44_ and Au_12_Ag_32_ cores, however, indicating that the added gold did not affect the ligand shell and crystal structure. This also indicates that the chemical stability can be improved with the addition of gold without changing the overall structure and mechanical properties of the MPC crystal.

The differences in the nature of the ligands were not found to have affected the overall arrangement of gold atoms in the MPC cores, with the gold atoms occupying the same positions in both structures. The different ligands induce slightly different bonding within the metal core, however. Bond lengths in the icosa­hedral core in Ag_44_ and Au_12_Ag_32_ are similar for both *p*-MBA and SPhF_2_ ligands, contracting 0.03 and 0.05 Å, respectively, with the addition of gold atoms. This indicates that changes in the icosa­hedral core are not influenced by the ligands. Changes in bond lengths are different in the dodeca­hedral core, however. In the case of *p*-MBA, bond lengths in the dodeca­hedron do not change with the addition of gold atoms, but in the case of the SPhF_2_ ligand they contract slightly. This indicates that changes in the dodeca­hedral core are influenced by the SPhF_2_ ligands, presumably due to their greater electron-withdrawing ability. The net effect is that the radius of the icosa­hedron contracts slightly in the case of both *p*-MBA and SPhF_2_ (0.03 and 0.05 Å, respectively), but the radius of the dodeca­hedron does not change for *p*-MBA while it contracts 0.03 Å in the case of SPhF_2_.

## Synthesis, crystallization, and theoretical methodology   

**Synthesis of** ***M*** **_4_Au_12_Ag_32_(*p*-MBA)_30_ by co-reduction**

*M*_4_Au_12_Ag_32_(*p*-MBA)_30_ MPCs were produced by first synthesizing a distribution of *M*_4_Au_*x*_Ag_44–*x*_(*p*-MBA)_30_ MPCs using an Au:Ag input ratio of 14:30. For this input ratio, 72.4 mg of AuCl_3_ (0.24 mmol) and 86.8 mg of AgNO_3_ (0.51 mmol) were used for the metal sources. These materials were added to 33 ml of 7:4 water–DMSO solvent along with 200 mg of *p*-MBA (1.3 mmol). This mixture was sonicated and stirred to fully dissolve the *p*-MBA. The dissolved *p*-MBA reacted with the metals to form a precursor mixture of metal thiol­ates, which was a cloudy light-yellow precipitate that was dispersed in the solvent. The pH was then adjusted to 12 using 50% *w*/*v* aqueous CsOH. The metal thiol­ates dissolved as the pH was raised above 9, forming a clear, light-yellow solution. Next, 5.0 mmol of NaBH_4_ reducing agent dissolved in 9 ml of water was added dropwise over a period of 30 min, and was then left to stir for 1 h. This formed a dark-yellow/brown solution. Once the reaction was completed, the product solution was centrifuged for 5 min (to remove insoluble byproducts), deca­nted, and then the supernatant was precip­itated using DMF. The precipitate was collected by centrifugation. It is important not to dry this raw product.

The raw product was precipitated from a basic solution; therefore it was the conjugate base (alkali metal salt) of the fully protonated species. To protonate, pure DMF was added to the precipitated particles, which did not initially solubilize. Glacial acetic acid was then added dropwise to the solution until the precipitate dissolved into the DMF, forming a golden brown solution. Protonation was repeated three times, using toluene to precipitate from DMF.

Fully protonated *M*_4_Au_*x*_Ag_44–*x*_(*p*-MBA)_30_ MPCs were next subjected to thermal processing. Capped glass vials containing DMF solutions of the MPCs were placed in a water bath at 333 K for 30 h. After incubation, insoluble material produced by thermal processing was separated from the solution by centrifugation. The supernatant was collected and was protonated with glacial acetic acid in DMF and precipitated with toluene. The protonation steps were repeated two times to ensure complete protonation of the carboxyl­ates. The fully protonated product enriched in *M*_4_Au_12_Ag_32_(*p*-MBA)_30_ was able to be dissolved in a neat solution of DMF.

With the above synthetic conditions, the counter-cations tend to be a mixture of alkali metals, namely Cs^+^ and Na^+^. The counter-ion mixture has been identified by energy dispersive X-ray spectroscopy (EDS) to be approximately a 3:1 ratio of Cs:Na, despite the expectation that there might be a higher affinity for Na because of its size. The mol­ecular formula for this MPC could therefore be written as NaCs_3_Au_12_Ag_32_(*p*-MBA)_30_. It should be noted, however, that the counter-ions are readily dissociated and easily exchanged such that the identities of the alkali metals play little role in the properties of the material. Nonetheless, Na_4_Au_12_Ag_32_(*p*-MBA)_30_ and K_4_Au_12_Ag_32_(*p*-MBA)_30_ can be directly prepared by using all-sodium and all-potassium reaction conditions, respectively, if desired (Desireddy *et al.*, 2013 ▸).

**Crystallization**

The *M*_4_Au_12_Ag_32_(*p*-MBA)_30_ crystals were grown from a neat DMF solution of MPCs, dried under N_2_ gas. Small rhombohedral crystals (10 µm) were obtained from this crystallization process. These crystals were used as seeds in a second crystallization step. The second solution was dried under N_2_ and the seeds grew into larger rhombohedral crystals (>50 µm). The crystals were first separated and isolated on a microscope slide using paratone oil, and then were picked up and mounted with a MiTeGen MicroLoop.

**Theoretical Methodology**

The density functional theory (DFT) calculations and Bader charge analysis (Bader, 1990 ▸; Tang *et al.*, 2009 ▸) were performed using the VASP–DFT package with a plane-wave basis with a kinetic energy cutoff of 400 eV, PAW pseudo­potentials (Kresse & Joubert, 1999 ▸), and the PW91 generalized gradient approximation (GGA) for the exchange-correlation potential (Perdew, 1991 ▸; Perdew *et al.*, 1992 ▸, 1993 ▸). For structure optimization, convergence was achieved for forces smaller than 0.001 eV Å^−1^. The X-ray determined structure of Na_4_Ag_44_(*p*-MBA)_30_ was taken as the starting configuration for structural relaxation. Hydrogen atoms were added to the structure and their positions were relaxed, yielding *d*(C—H) = 1.09 Å.

To estimate the inter-ligand van der Waals (vdW) inter­action energy, the total energy of the relaxed Na_4_Au_12_Ag_32_(*p-*MBA)_30_ MPC was evaluated with and without the inclusion of the vdW inter­actions, using density functional theory (DFT) (Grimme, 2006 ▸). The energy of the MPC, calculated with the inclusion of the vdW inter­actions between the atomic constit­uents of the ligand (S, C, O and H atoms) was found to be lower by Δ_tot_ (vdW) = 13.23 eV compared to that found without the inclusion of the vdW contributions. However, this vdW energy includes intra­molecular and inter­molecular inter­actions between the ligand mol­ecules. The average intra-ligand (Ag—S—C_6_H_4_—COOH) vdW stabilization energy was calculated (for the relaxed configuration of the Ag-bonded ligand mol­ecule) using DFT to be Δ_intra_ (vdW) = 0.251 eV. The total inter­molecular vdW energy in the ligand shell (made of 30 *p*-MBA mol­ecules) is therefore calculated as: Δ_inter_ (vdW) = Δ_tot_ (vdW) − 30 Δ_intra_ (vdW) = 5.70 eV. Since the ligand mol­ecules are assembled into six Ag_2_(*p-*MBA)_5_ mounts, we conclude that the inter-ligand non-bonded (dispersion, vdW) energy is 0.95 eV/mount.

## Refinement   

All of the Ag, Au and S atoms were located by direct methods. During the following refinements and subsequent difference-Fourier syntheses, the remaining C atoms and O atoms were located.

The Au, Ag and S atoms were ordered; however three out of the five crystallographically independent ligands in the asymmetric unit cell were disordered over two sets of sites. The three disordered ligands were modeled over the two positions, and their occupancies were refined with fixed atomic displacement parameters using a free variable to be 0.5. Final refinement released the fixed atomic displacement parameter and constrained the occupancies to be 0.5 for all disordered C and O atoms.

Au, Ag, and S atoms were refined with anisotropic displace­ment parameters, while all C and O atoms were refined with isotropic atomic displacement parameters. DFIX restraints were applied to the C—O bonds in the carb­oxy­lic acid groups, but C-atom positions in the phenyl rings were not restrained. All H atoms were geometrically determined on idealized positions (O—H = 0.84, C—H = 0.95°), using AFIX 43 and AFIX 83 instructions, and were included as riding atoms in the final refinements [*U*_iso_(H) = 1.2*U*_eq_(C) or 1.5*U*_eq_(O)].

It is common for MPCs to have a high amount of residual electron density observed in the metal core. It is noted that the alkali metal cations and the solvent mol­ecules were not identified in the X-ray data (highest residue density was 2.55 e Å^−3^). *PLATON* (Spek, 2009 ▸) was used to determine the total void volume in the unit cell to be about 52% with an estimate of 19000 electrons. Attempts to improve the refinement using the SQUEEZE (Spek, 2015 ▸) option in *PLATON* were not successful.

Crystal data, data collection and structure refinement details are summarized in Table 3 ▸. Note that the given formula, density, *etc*. in this Table refers to the refined part of the structure and do not include the type of counter ions and solvent mol­ecules.

## Supplementary Material

Crystal structure: contains datablock(s) I. DOI: 10.1107/S2056989018008393/wm5444sup1.cif

Structure factors: contains datablock(s) I. DOI: 10.1107/S2056989018008393/wm5444Isup2.hkl

CCDC reference: 1847794

Additional supporting information:  crystallographic information; 3D view; checkCIF report

## Figures and Tables

**Table 1:** Comparison of metal–metal bond lengths (Å) in *M*_4_Ag_44_(*p*-MBA)_30_, *M*_4_Au_12_Ag_32_(*p*-MBA)_30_, (PPh_4_)_4_Ag_44_(SPhF_2_)_30_ and (PPh_4_)_4_Au_12_Ag_32_(SPhF_2_)_30_, with standard deviations

	*M*_4_Ag_44_(*p*-MBA)_30_	*M*_4_Au_12_Ag_32_(*p*-MBA)_30_	(PPh_4_)_4_Ag_44_(SPhF_2_)_30_	(PPh_4_)_4_Au_12_Ag_32_(SPhF_2_)_30_
12-Atom icosa­hedron	2.825 ± 0.012	2.795 ± 0.013	2.831 ± 0.019	2.779 ± 0.018
20-Atom dodeca­hedron	3.175 ± 0.040	3.190 ± 0.040	3.167 ± 0.088	3.151 ± 0.066
Icosa­hedron radius	2.688 ± 0.005	2.659 ± 0.009	2.691 ± 0.018	2.644 ± 0.013
Dodeca­hedron radius	4.461 ± 0.021	4.461 ± 0.020	4.468 ± 0.032	4.436 ± 0.029
Ag—Ag in mounts	2.995 ± 0.001	2.992 ± 0.001	2.973 ± 0.016	2.945 ± 0.014

**Table 2:** Bader analysis results showing excess electrons, Δn_e_, with per-atom values listed in parentheses Δ*n*_e_ > 0 corresponds to excess electrons (negative charge accumulation) and Δ*n*_e_ < 0 corresponds to electron depletion (positive charge accumulation).

	*M*_4_Ag_44_(*p*-MBA)_30_	*M*_4_Au_12_Ag_32_(*p*-MBA)_30_
Δ*n*_e_(icosa­hedron)	0.010 (0.001)	1.769 (0.147)
Δ*n*_e_(dodeca­hedron)	−4.928 (−0.246)	−6.546 (−0.327)
Δ*n*_e_(mounts)	−4.095 (−0.341)	−4.106 (−0.342)

**Table 3:** Experimental details

Crystal data	
Chemical formula	Ag_32_Au_12_(C_7_H_5_O_2_S)_30_
*M*_r_	10410.53
Crystal system, space group	Trigonal, *R* 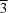 *c*:*H*
Temperature (K)	100
*a*, *c* (Å)	25.7341 (3), 124.079 (4)
*V* (Å^3^)	71162 (3)
*Z*	6
Radiation type	Cu *K*α
μ (mm^−1^)	18.65
Crystal size (mm)	0.2 × 0.2 × 0.1

Data collection	
Diffractometer	Bruker APEX Duo CCD
Absorption correction	Multi-scan (*SADABS*; Sheldrick, 1996 ▸)
*T*_min_, *T*_max_	0.565, 0.752
No. of measured, independent and observed [*I* > 2σ(*I*)] reflections	182413, 12622, 11138
*R*_int_	0.055
θ_max_ (°)	62.4
(sin θ/λ)_max_ (Å^−1^)	0.575

Refinement	
*R*[*F* ^2^ > 2σ(*F* ^2^)], *wR*(*F* ^2^), *S*	0.040, 0.139, 1.09
No. of reflections	12622
No. of parameters	364
No. of restraints	16
H-atom treatment	H-atom parameters constrained
Δρ_max_, Δρ_min_ (e Å^−3^)	2.54, −1.52

Computer programs: *APEX2* and *SAINT* (Bruker, 2012 ▸), *SHELXS* (Sheldrick, 2008 ▸), *SHELXL2014/7* (Sheldrick, 2015 ▸), *Mercury* (Macrae *et al.*, 2008 ▸) and *publCIF* (Westrip, 2010 ▸).

## supplementary crystallographic information

### Crystal data

**Table:**

Ag_32_Au_12_(C_7_H_5_O_2_S)_30_	*D*_x_ = 1.458 Mg m^−^^3^
*M**_r_* = 10410.53	Cu *K*α radiation, λ = 1.54178 Å
Trigonal, *R*3*c*:*H*	Cell parameters from 9758 reflections
*a* = 25.7341 (3) Å	θ = 4.1–62.4°
*c* = 124.079 (4) Å	µ = 18.65 mm^−^^1^
*V* = 71162 (3) Å^3^	*T* = 100 K
*Z* = 6	Rhombohedral, dark_purple
*F*(000) = 28932	0.2 × 0.2 × 0.1 mm

### Data collection

**Table:**

Bruker APEX Duo CCD diffractometer	11138 reflections with *I* > 2σ(*I*)
Radiation source: ImuS	*R*_int_ = 0.055
phi and ω scans	θ_max_ = 62.4°, θ_min_ = 2.1°
Absorption correction: multi-scan (SADABS; Sheldrick, 1996)	*h* = −29→28
*T*_min_ = 0.565, *T*_max_ = 0.752	*k* = −29→29
182413 measured reflections	*l* = −134→142
12622 independent reflections	

### Refinement

**Table:**

Refinement on *F*^2^	Primary atom site location: structure-invariant direct methods
Least-squares matrix: full	Secondary atom site location: difference Fourier map
*R*[*F*^2^ > 2σ(*F*^2^)] = 0.040	Hydrogen site location: inferred from neighbouring sites
*wR*(*F*^2^) = 0.139	H-atom parameters constrained
*S* = 1.09	*w* = 1/[σ^2^(*F*_o_^2^) + (0.0801*P*)^2^ + 2212.5481*P*] where *P* = (*F*_o_^2^ + 2*F*_c_^2^)/3
12622 reflections	(Δ/σ)_max_ < 0.001
364 parameters	Δρ_max_ = 2.54 e Å^−^^3^
16 restraints	Δρ_min_ = −1.52 e Å^−^^3^

### Special details

**Table:**

Geometry. All esds (except the esd in the dihedral angle between two l.s. planes) are estimated using the full covariance matrix. The cell esds are taken into account individually in the estimation of esds in distances, angles and torsion angles; correlations between esds in cell parameters are only used when they are defined by crystal symmetry. An approximate (isotropic) treatment of cell esds is used for estimating esds involving l.s. planes.
Refinement. The data reported herein were collected from a crystal of approximate dimensions 200 x 200 x 100 µm^3^, which was cooled to 100 K for data collection. X-ray diffraction data were collected on a Bruker Apex Duo diffractometer (CuKα = 1.54178 Å), which was equipped with an Apex II CCD detector and an Oxford Cryostream 700 low temperature device.The frames were integrated with the Bruker SAINT software package using a narrow-frame algorithm. The integration of the data using a trigonal unit cell yielded a total of 182413 reflections to a maximum θ angle of 62.42° (0.87 Å resolution), of which 12622 were independent (average redundancy 14.452, completeness = 100.0%, R_int_ = 5.49%, R_sig_ = 1.92%) and 11138 (88.24%) were greater than 2σ(F^2^). The final cell constants of a = 25.7341 (3) Å, b = 25.7341 (3) Å, c = 124.079 (4) Å, volume = 71162 (3) Å^3^, are based upon the refinement of the XYZ-centroids of reflections above 20 σ(I).The structure was solved and refined using the Bruker SHELXTL software package (Sheldrick, 2015), using the *R*3*c* space group, with Z = 6 for the formula unit C_210_H_150_Ag_32_Au_12_O_60_S_30_. The final full-matrix least-squares refinement on F^2^ with 373 variables converged at R1 = 3.98% for the observed data and wR2 = 13.91% for all data. The goodness-of-fit was 1.094. The largest peak in the final difference electron density synthesis was 2.547 e^-^/Å^3^ and the largest hole was -1.528 e/Å^3^ with an RMS deviation of 0.253 e/Å^3^. On the basis of the final model, the calculated density was 1.458 g/cm^3^ and F(000) was 28932 e^-^. The cations and solvent molecules are not included in the calculations of the density or F(000).

### Fractional atomic coordinates and isotropic or equivalent isotropic displacement parameters (Å^2^)

**Table:**

	*x*	*y*	*z*	*U*_iso_*/*U*_eq_	Occ. (<1)
Au1	0.04843 (2)	0.07066 (2)	0.01714 (2)	0.01581 (10)	
Au2	0.07933 (2)	0.11391 (2)	−0.00404 (2)	0.01596 (10)	
Ag1	0.06659 (3)	0.18648 (2)	0.01209 (2)	0.02133 (14)	
Ag2	0.16900 (3)	0.15380 (3)	0.01221 (2)	0.02476 (15)	
Ag3	0.12936 (3)	0.04066 (3)	0.02703 (2)	0.02510 (15)	
Ag4	0.0000	0.0000	0.03625 (2)	0.0219 (2)	
Ag5	0.26323 (3)	0.12998 (4)	0.02307 (2)	0.0454 (2)	
Ag6	0.19339 (4)	0.16795 (3)	0.03705 (2)	0.0446 (2)	
S1	0.10178 (11)	0.26224 (10)	−0.00377 (2)	0.0339 (5)	
S2	0.15995 (10)	0.22519 (10)	0.02506 (2)	0.0338 (5)	
S3	0.21203 (11)	0.01710 (11)	0.02284 (2)	0.0352 (5)	
S4	0.10443 (11)	0.07711 (11)	0.04443 (2)	0.0340 (5)	
S5	0.30419 (15)	0.21218 (16)	0.03686 (3)	0.0678 (9)	
O1	0.0603 (9)	0.4829 (9)	0.01510 (15)	0.159 (7)*	
O2	0.0670 (8)	0.5043 (9)	−0.00219 (14)	0.156 (6)*	
H2B	0.0854	0.5411	−0.0008	0.234*	
O3	0.3977 (8)	0.4646 (8)	0.00301 (15)	0.152 (6)*	
O4	0.4186 (9)	0.4802 (9)	0.02040 (16)	0.182 (8)*	
H30	0.4525	0.5044	0.0179	0.273*	
C1	0.0892 (5)	0.3229 (5)	−0.00131 (9)	0.044 (2)*	
C2	0.0840 (7)	0.3366 (8)	0.00909 (14)	0.083 (4)*	
H2	0.0844	0.3127	0.0149	0.099*	
C3	0.0782 (9)	0.3862 (10)	0.01093 (18)	0.112 (6)*	
H3	0.0742	0.3958	0.0182	0.134*	
C4	0.0777 (8)	0.4212 (8)	0.00311 (15)	0.089 (5)*	
C5	0.0775 (9)	0.4038 (9)	−0.00702 (17)	0.104 (6)*	
H5	0.0712	0.4248	−0.0127	0.125*	
C6	0.0861 (7)	0.3567 (8)	−0.00948 (14)	0.085 (5)*	
H6	0.0898	0.3477	−0.0168	0.101*	
C7	0.0730 (11)	0.4749 (11)	0.00580 (16)	0.126 (8)*	
C8	0.2244 (5)	0.2937 (5)	0.02136 (9)	0.042 (2)*	
C9	0.2413 (7)	0.3085 (7)	0.01055 (13)	0.076 (4)*	
H9	0.2180	0.2805	0.0051	0.092*	
C10	0.2905 (9)	0.3621 (9)	0.00755 (17)	0.105 (6)*	
H10	0.3002	0.3727	0.0002	0.125*	
C11	0.3264 (8)	0.4012 (8)	0.01608 (15)	0.088 (5)*	
C12	0.3145 (10)	0.3810 (10)	0.02632 (19)	0.119 (7)*	
H12	0.3423	0.4042	0.0318	0.143*	
C13	0.2630 (8)	0.3273 (8)	0.02929 (16)	0.093 (5)*	
H13	0.2553	0.3149	0.0366	0.112*	
C14	0.3790 (10)	0.4579 (11)	0.01247 (17)	0.128 (8)*	
C15	0.2296 (5)	−0.0106 (5)	0.03434 (9)	0.042 (2)*	
C16A	0.2830 (12)	−0.0094 (12)	0.0343 (2)	0.062 (7)*	0.5
H16	0.3093	0.0063	0.0283	0.074*	0.5
C17A	0.2987 (13)	−0.0318 (13)	0.0433 (2)	0.066 (7)*	0.5
H17	0.3363	−0.0304	0.0434	0.079*	0.5
C16B	0.2063 (11)	−0.0699 (11)	0.03674 (19)	0.052 (6)*	0.5
H15A	0.1767	−0.0981	0.0320	0.062*	0.5
C17B	0.2215 (12)	−0.0920 (13)	0.0452 (2)	0.061 (7)*	0.5
H16D	0.2029	−0.1341	0.0463	0.073*	0.5
C18	0.2618 (7)	−0.0556 (7)	0.05210 (12)	0.070 (4)*	
C19A	0.2032 (13)	−0.0594 (13)	0.0516 (2)	0.068 (7)*	0.5
H16A	0.1761	−0.0763	0.0575	0.081*	0.5
C20A	0.1868 (12)	−0.0387 (11)	0.0427 (2)	0.058 (6)*	0.5
H15	0.1479	−0.0431	0.0422	0.069*	0.5
C19B	0.292 (2)	0.009 (2)	0.0505 (4)	0.116 (14)*	0.5
H19A	0.3232	0.0364	0.0551	0.139*	0.5
C20B	0.2722 (15)	0.0296 (15)	0.0417 (3)	0.079 (8)*	0.5
H19B	0.2882	0.0715	0.0407	0.095*	0.5
C21	0.2809 (8)	−0.0758 (8)	0.06148 (14)	0.088 (5)*	
O5A	0.3244 (11)	−0.0827 (12)	0.0607 (2)	0.106 (8)*	0.5
O6A	0.2398 (11)	−0.1019 (13)	0.0689 (2)	0.115 (9)*	0.5
H6A2	0.2368	−0.0757	0.0725	0.172*	0.5
O5B	0.2560 (10)	−0.1306 (8)	0.06321 (18)	0.087 (6)*	0.5
O6B	0.3251 (11)	−0.0364 (11)	0.0677 (2)	0.116 (9)*	0.5
H5B2	0.3582	−0.0269	0.0648	0.173*	0.5
C22	0.1284 (5)	0.0498 (5)	0.05536 (9)	0.042 (2)*	
C23A	0.1932 (14)	0.0827 (14)	0.0578 (3)	0.076 (8)*	0.5
H23	0.2198	0.1171	0.0537	0.091*	0.5
C24A	0.2140 (15)	0.0630 (15)	0.0659 (3)	0.079 (9)*	0.5
H24	0.2558	0.0816	0.0673	0.095*	0.5
C23B	0.1297 (12)	−0.0051 (12)	0.0543 (2)	0.063 (7)*	0.5
H23D	0.1176	−0.0272	0.0477	0.075*	0.5
C24B	0.1490 (14)	−0.0256 (14)	0.0630 (2)	0.073 (8)*	0.5
H24D	0.1485	−0.0627	0.0627	0.087*	0.5
C25	0.1696 (7)	0.0115 (7)	0.07240 (13)	0.072 (4)*	
C26A	0.1127 (12)	−0.0137 (12)	0.0701 (2)	0.061 (7)*	0.5
H26	0.0847	−0.0461	0.0744	0.074*	0.5
C27A	0.0920 (11)	0.0051 (11)	0.06161 (19)	0.052 (6)*	0.5
H27	0.0501	−0.0148	0.0602	0.062*	0.5
C26B	0.1633 (15)	0.0579 (15)	0.0734 (3)	0.079 (9)*	0.5
H26D	0.1729	0.0789	0.0800	0.095*	0.5
C27B	0.1424 (14)	0.0778 (14)	0.0648 (2)	0.074 (8)*	0.5
H27D	0.1382	0.1122	0.0657	0.089*	0.5
C28	0.1937 (8)	−0.0066 (7)	0.08157 (14)	0.087 (5)*	
O7A	0.1580 (9)	−0.0490 (9)	0.08716 (17)	0.081 (6)*	0.5
O8A	0.2526 (10)	0.0242 (11)	0.0834 (2)	0.114 (9)*	0.5
H7AB	0.2711	0.0271	0.0776	0.171*	0.5
O7B	0.2052 (13)	0.0231 (12)	0.09017 (19)	0.115 (9)*	0.5
O8B	0.1907 (13)	−0.0591 (10)	0.0807 (2)	0.113 (8)*	0.5
H7BC	0.2241	−0.0540	0.0787	0.170*	0.5
C29	0.3289 (7)	0.1857 (7)	0.04713 (13)	0.075 (4)*	
C30A	0.3335 (16)	0.1336 (16)	0.0455 (3)	0.087 (9)*	0.5
H30D	0.3222	0.1136	0.0387	0.104*	0.5
C31A	0.3559 (18)	0.1093 (19)	0.0543 (3)	0.102 (11)*	0.5
H31	0.3580	0.0736	0.0536	0.122*	0.5
C30B	0.2911 (17)	0.1600 (15)	0.0569 (3)	0.087 (9)*	0.5
H30A	0.2534	0.1582	0.0574	0.104*	0.5
C31B	0.311 (2)	0.1388 (19)	0.0654 (4)	0.112 (13)*	0.5
H31A	0.2864	0.1197	0.0715	0.134*	0.5
C32	0.3749 (9)	0.1477 (9)	0.06437 (16)	0.101 (6)*	
C33A	0.372 (2)	0.199 (2)	0.0655 (4)	0.118 (14)*	0.5
H32	0.3863	0.2225	0.0719	0.141*	0.5
C34A	0.3480 (17)	0.2182 (18)	0.0574 (3)	0.096 (11)*	0.5
H34	0.3441	0.2526	0.0584	0.115*	0.5
C33B	0.4124 (19)	0.1760 (17)	0.0556 (3)	0.101 (11)*	0.5
H32A	0.4510	0.1798	0.0551	0.121*	0.5
C34B	0.3891 (16)	0.2002 (16)	0.0471 (3)	0.089 (10)*	0.5
H34A	0.4154	0.2256	0.0417	0.107*	0.5
C35	0.3961 (10)	0.1233 (11)	0.07298 (18)	0.118 (7)*	
O9A	0.4219 (14)	0.1564 (14)	0.0809 (2)	0.133 (10)*	0.5
O10A	0.4058 (18)	0.0792 (14)	0.0704 (3)	0.161 (13)*	0.5
H10B	0.4388	0.0860	0.0730	0.241*	0.5
O9B	0.3657 (13)	0.1071 (13)	0.0814 (2)	0.119 (9)*	0.5
O10B	0.4520 (12)	0.1357 (16)	0.0722 (3)	0.140 (11)*	0.5
H10C	0.4745	0.1681	0.0754	0.210*	0.5

### Atomic displacement parameters (Å^2^)

**Table:**

	*U*^11^	*U*^22^	*U*^33^	*U*^12^	*U*^13^	*U*^23^
Au1	0.01673 (17)	0.01546 (17)	0.01451 (17)	0.00750 (13)	−0.00106 (12)	−0.00080 (12)
Au2	0.01646 (17)	0.01459 (17)	0.01613 (18)	0.00724 (13)	0.00094 (12)	0.00051 (12)
Ag1	0.0255 (3)	0.0142 (3)	0.0232 (3)	0.0091 (2)	−0.0015 (2)	−0.0036 (2)
Ag2	0.0195 (3)	0.0196 (3)	0.0253 (3)	0.0024 (2)	−0.0057 (2)	0.0001 (2)
Ag3	0.0236 (3)	0.0258 (3)	0.0217 (3)	0.0092 (3)	−0.0089 (2)	0.0015 (2)
Ag4	0.0269 (3)	0.0269 (3)	0.0119 (5)	0.01346 (17)	0.000	0.000
Ag5	0.0306 (4)	0.0455 (4)	0.0491 (4)	0.0109 (3)	−0.0096 (3)	0.0091 (4)
Ag6	0.0528 (5)	0.0344 (4)	0.0340 (4)	0.0124 (4)	−0.0132 (3)	−0.0023 (3)
S1	0.0498 (14)	0.0208 (11)	0.0338 (12)	0.0198 (10)	0.0045 (10)	0.0033 (9)
S2	0.0291 (11)	0.0241 (11)	0.0347 (12)	0.0033 (9)	−0.0099 (9)	−0.0027 (9)
S3	0.0331 (12)	0.0380 (13)	0.0358 (12)	0.0189 (11)	−0.0149 (10)	0.0000 (10)
S4	0.0337 (12)	0.0387 (13)	0.0216 (11)	0.0120 (10)	−0.0104 (9)	0.0013 (9)
S5	0.0530 (18)	0.062 (2)	0.065 (2)	0.0111 (16)	−0.0263 (16)	−0.0033 (16)

### Geometric parameters (Å, º)

**Table:**

Au1—Au2^i^	2.7802 (4)	C11—C12	1.35 (3)
Au1—Au1^ii^	2.7893 (5)	C11—C14	1.48 (3)
Au1—Au1^iii^	2.7893 (5)	C12—C13	1.40 (3)
Au1—Au2	2.8089 (4)	C12—H12	0.9500
Au1—Au2^iv^	2.8115 (4)	C13—H13	0.9500
Au1—Ag2	2.8179 (7)	C15—C16A	1.36 (3)
Au1—Ag3^ii^	2.8362 (7)	C15—C16B	1.36 (3)
Au1—Ag3	2.8372 (7)	C15—C20B	1.40 (3)
Au1—Ag1	2.8462 (6)	C15—C20A	1.42 (3)
Au1—Ag4	2.8664 (8)	C16A—C17A	1.41 (4)
Au2—Au1^iv^	2.7804 (4)	C16A—H16	0.9500
Au2—Au2^i^	2.7897 (4)	C17A—C18	1.37 (3)
Au2—Au2^iv^	2.7898 (4)	C17A—H17	0.9500
Au2—Au1^i^	2.8117 (4)	C16B—C17B	1.35 (3)
Au2—Ag2	2.8414 (6)	C16B—H15A	0.9500
Au2—Ag2^i^	2.8459 (7)	C17B—C18	1.31 (3)
Au2—Ag1^iv^	2.8639 (6)	C17B—H16D	0.9500
Au2—Ag1	2.8661 (6)	C18—C21	1.46 (2)
Au2—Ag3^i^	2.8736 (7)	C18—C19B	1.46 (5)
Ag1—S1	2.594 (2)	C18—C19A	1.46 (3)
Ag1—S3^ii^	2.633 (2)	C19A—C20A	1.38 (4)
Ag1—S2	2.638 (2)	C19A—H16A	0.9500
Ag1—Au2^i^	2.8637 (6)	C20A—H15	0.9500
Ag1—Ag2	3.1417 (9)	C19B—C20B	1.41 (5)
Ag1—Ag3^ii^	3.2309 (9)	C19B—H19A	0.9500
Ag1—Ag2^i^	3.2323 (8)	C20B—H19B	0.9500
Ag2—S2	2.530 (3)	C21—O5A	1.222 (17)
Ag2—S1^iv^	2.544 (2)	C21—O5B	1.240 (16)
Ag2—Au2^iv^	2.8457 (7)	C21—O6A	1.308 (17)
Ag2—Ag5	3.0921 (10)	C21—O6B	1.328 (17)
Ag2—Ag6	3.1309 (9)	O6A—H6A2	0.8400
Ag2—Ag3	3.1516 (8)	O6B—H5B2	0.8400
Ag2—Ag1^iv^	3.2320 (8)	C22—C27A	1.31 (3)
Ag3—S3	2.540 (3)	C22—C27B	1.33 (3)
Ag3—S4	2.560 (2)	C22—C23B	1.43 (3)
Ag3—Au1^iii^	2.8364 (7)	C22—C23A	1.48 (3)
Ag3—Au2^iv^	2.8737 (7)	C23A—C24A	1.35 (4)
Ag3—Ag5	3.0781 (10)	C23A—H23	0.9500
Ag3—Ag6	3.0973 (10)	C24A—C25	1.49 (4)
Ag3—Ag4	3.1624 (7)	C24A—H24	0.9500
Ag3—Ag1^iii^	3.2313 (9)	C23B—C24B	1.40 (4)
Ag4—S4^ii^	2.619 (2)	C23B—H23D	0.9500
Ag4—S4	2.619 (2)	C24B—C25	1.43 (3)
Ag4—S4^iii^	2.619 (2)	C24B—H24D	0.9500
Ag4—Au1^ii^	2.8663 (8)	C25—C26B	1.29 (3)
Ag4—Au1^iii^	2.8663 (8)	C25—C26A	1.30 (3)
Ag4—Ag3^iii^	3.1625 (7)	C25—C28	1.48 (2)
Ag4—Ag3^ii^	3.1625 (7)	C26A—C27A	1.37 (3)
Ag5—S5	2.506 (4)	C26A—H26	0.9500
Ag5—S3	2.519 (3)	C27A—H27	0.9500
Ag5—S1^iv^	2.525 (2)	C26B—C27B	1.40 (4)
Ag5—Ag6	2.9916 (13)	C26B—H26D	0.9500
Ag6—S5	2.486 (4)	C27B—H27D	0.9500
Ag6—S4	2.489 (2)	C28—O7A	1.231 (16)
Ag6—S2	2.528 (2)	C28—O7B	1.259 (17)
S1—C1	1.773 (11)	C28—O8B	1.319 (17)
S1—Ag5^i^	2.524 (2)	C28—O8A	1.333 (17)
S1—Ag2^i^	2.544 (2)	O8A—H7AB	0.8400
S2—C8	1.774 (11)	O8B—H7BC	0.8400
S3—C15	1.753 (11)	C29—C34B	1.40 (4)
S3—Ag1^iii^	2.633 (2)	C29—C30A	1.42 (4)
S4—C22	1.774 (11)	C29—C34A	1.46 (4)
S5—C29	1.711 (16)	C29—C30B	1.49 (4)
O1—C7	1.244 (16)	C30A—C31A	1.51 (5)
O2—C7	1.303 (16)	C30A—H30D	0.9500
O2—H2B	0.8400	C31A—C32	1.51 (4)
O3—C14	1.247 (16)	C31A—H31	0.9500
O4—C14	1.323 (16)	C30B—C31B	1.39 (5)
O4—H30	0.8400	C30B—H30A	0.9500
C1—C2	1.362 (19)	C31B—C32	1.55 (5)
C1—C6	1.36 (2)	C31B—H31A	0.9500
C2—C3	1.38 (2)	C32—C33A	1.38 (5)
C2—H2	0.9500	C32—C33B	1.39 (4)
C3—C4	1.33 (3)	C32—C35	1.48 (3)
C3—H3	0.9500	C33A—C34A	1.39 (5)
C4—C5	1.33 (2)	C33A—H32	0.9500
C4—C7	1.48 (3)	C34A—H34	0.9500
C5—C6	1.37 (2)	C33B—C34B	1.49 (5)
C5—H5	0.9500	C33B—H32A	0.9500
C6—H6	0.9500	C34B—H34A	0.9500
C8—C13	1.36 (2)	C35—O9B	1.246 (17)
C8—C9	1.403 (18)	C35—O9A	1.250 (18)
C9—C10	1.38 (2)	C35—O10B	1.311 (18)
C9—H9	0.9500	C35—O10A	1.318 (18)
C10—C11	1.43 (3)	O10A—H10B	0.8400
C10—H10	0.9500	O10B—H10C	0.8400
			
Au2^i^—Au1—Au1^ii^	60.638 (12)	Ag3—Ag4—Ag3^iii^	107.70 (2)
Au2^i^—Au1—Au1^iii^	108.352 (11)	S4^ii^—Ag4—Ag3^ii^	51.53 (6)
Au1^ii^—Au1—Au1^iii^	60.0	S4—Ag4—Ag3^ii^	100.24 (5)
Au2^i^—Au1—Au2	59.883 (8)	S4^iii^—Ag4—Ag3^ii^	149.88 (6)
Au1^ii^—Au1—Au2	108.158 (10)	Au1^ii^—Ag4—Ag3^ii^	55.884 (16)
Au1^iii^—Au1—Au2	107.485 (10)	Au1^iii^—Ag4—Ag3^ii^	102.99 (3)
Au2^i^—Au1—Au2^iv^	107.480 (16)	Au1—Ag4—Ag3^ii^	55.865 (16)
Au1^ii^—Au1—Au2^iv^	107.468 (11)	Ag3—Ag4—Ag3^ii^	107.70 (2)
Au1^iii^—Au1—Au2^iv^	59.522 (12)	Ag3^iii^—Ag4—Ag3^ii^	107.69 (2)
Au2—Au1—Au2^iv^	59.519 (8)	S5—Ag5—S3	137.53 (10)
Au2^i^—Au1—Ag2	114.254 (17)	S5—Ag5—S1^iv^	116.78 (10)
Au1^ii^—Au1—Ag2	166.301 (15)	S3—Ag5—S1^iv^	105.47 (8)
Au1^iii^—Au1—Ag2	113.81 (2)	S5—Ag5—Ag6	52.88 (9)
Au2—Au1—Ag2	60.660 (15)	S3—Ag5—Ag6	109.26 (7)
Au2^iv^—Au1—Ag2	60.730 (16)	S1^iv^—Ag5—Ag6	110.33 (7)
Au2^i^—Au1—Ag3^ii^	61.531 (15)	S5—Ag5—Ag3	111.27 (9)
Au1^ii^—Au1—Ag3^ii^	60.566 (17)	S3—Ag5—Ag3	52.84 (6)
Au1^iii^—Au1—Ag3^ii^	114.125 (17)	S1^iv^—Ag5—Ag3	101.65 (5)
Au2—Au1—Ag3^ii^	115.587 (17)	Ag6—Ag5—Ag3	61.35 (2)
Au2^iv^—Au1—Ag3^ii^	166.218 (18)	S5—Ag5—Ag2	99.84 (9)
Ag2—Au1—Ag3^ii^	130.01 (2)	S3—Ag5—Ag2	102.34 (6)
Au2^i^—Au1—Ag3	166.617 (18)	S1^iv^—Ag5—Ag2	52.68 (6)
Au1^ii^—Au1—Ag3	114.098 (16)	Ag6—Ag5—Ag2	61.92 (2)
Au1^iii^—Au1—Ag3	60.538 (17)	Ag3—Ag5—Ag2	61.43 (2)
Au2—Au1—Ag3	114.325 (17)	S5—Ag6—S4	137.68 (11)
Au2^iv^—Au1—Ag3	61.156 (15)	S5—Ag6—S2	111.98 (10)
Ag2—Au1—Ag3	67.738 (18)	S4—Ag6—S2	110.04 (8)
Ag3^ii^—Au1—Ag3	128.36 (3)	S5—Ag6—Ag5	53.50 (9)
Au2^i^—Au1—Ag1	61.177 (15)	S4—Ag6—Ag5	109.10 (7)
Au1^ii^—Au1—Ag1	115.60 (2)	S2—Ag6—Ag5	106.52 (6)
Au1^iii^—Au1—Ag1	166.782 (13)	S5—Ag6—Ag3	111.24 (10)
Au2—Au1—Ag1	60.900 (14)	S4—Ag6—Ag3	53.21 (6)
Au2^iv^—Au1—Ag1	114.024 (17)	S2—Ag6—Ag3	102.95 (6)
Ag2—Au1—Ag1	67.374 (19)	Ag5—Ag6—Ag3	60.70 (2)
Ag3^ii^—Au1—Ag1	69.302 (19)	S5—Ag6—Ag2	99.27 (9)
Ag3—Au1—Ag1	128.43 (2)	S4—Ag6—Ag2	102.49 (5)
Au2^i^—Au1—Ag4	115.242 (13)	S2—Ag6—Ag2	51.79 (6)
Au1^ii^—Au1—Ag4	60.885 (10)	Ag5—Ag6—Ag2	60.62 (2)
Au1^iii^—Au1—Ag4	60.884 (10)	Ag3—Ag6—Ag2	60.80 (2)
Au2—Au1—Ag4	166.498 (17)	C1—S1—Ag5^i^	110.7 (4)
Au2^iv^—Au1—Ag4	114.256 (13)	C1—S1—Ag2^i^	116.3 (4)
Ag2—Au1—Ag4	128.732 (18)	Ag5^i^—S1—Ag2^i^	75.20 (7)
Ag3^ii^—Au1—Ag4	67.360 (15)	C1—S1—Ag1	112.5 (4)
Ag3—Au1—Ag4	67.343 (14)	Ag5^i^—S1—Ag1	135.83 (9)
Ag1—Au1—Ag4	129.626 (18)	Ag2^i^—S1—Ag1	77.96 (7)
Au1^iv^—Au2—Au2^i^	108.283 (10)	C8—S2—Ag6	108.0 (4)
Au1^iv^—Au2—Au2^iv^	60.575 (9)	C8—S2—Ag2	100.3 (4)
Au2^i^—Au2—Au2^iv^	107.825 (12)	Ag6—S2—Ag2	76.48 (7)
Au1^iv^—Au2—Au1	108.883 (15)	C8—S2—Ag1	116.1 (4)
Au2^i^—Au2—Au1	59.547 (12)	Ag6—S2—Ag1	130.54 (9)
Au2^iv^—Au2—Au1	60.287 (12)	Ag2—S2—Ag1	74.83 (6)
Au1^iv^—Au2—Au1^i^	59.836 (14)	C15—S3—Ag5	111.5 (4)
Au2^i^—Au2—Au1^i^	60.201 (9)	C15—S3—Ag3	110.3 (4)
Au2^iv^—Au2—Au1^i^	108.071 (10)	Ag5—S3—Ag3	74.94 (7)
Au1—Au2—Au1^i^	107.996 (15)	C15—S3—Ag1^iii^	112.9 (4)
Au1^iv^—Au2—Ag2	115.290 (18)	Ag5—S3—Ag1^iii^	133.37 (9)
Au2^i^—Au2—Ag2	113.22 (2)	Ag3—S3—Ag1^iii^	77.28 (6)
Au2^iv^—Au2—Ag2	60.701 (17)	C22—S4—Ag6	108.3 (4)
Au1—Au2—Ag2	59.826 (15)	C22—S4—Ag3	107.4 (4)
Au1^i^—Au2—Ag2	165.951 (18)	Ag6—S4—Ag3	75.67 (7)
Au1^iv^—Au2—Ag2^i^	113.215 (17)	C22—S4—Ag4	115.1 (4)
Au2^i^—Au2—Ag2^i^	60.555 (16)	Ag6—S4—Ag4	133.05 (9)
Au2^iv^—Au2—Ag2^i^	165.734 (16)	Ag3—S4—Ag4	75.26 (6)
Au1—Au2—Ag2^i^	114.251 (17)	C29—S5—Ag6	112.3 (6)
Au1^i^—Au2—Ag2^i^	59.743 (15)	C29—S5—Ag5	104.8 (6)
Ag2—Au2—Ag2^i^	129.876 (18)	Ag6—S5—Ag5	73.62 (9)
Au1^iv^—Au2—Ag1^iv^	60.548 (14)	C7—O2—H2B	109.5
Au2^i^—Au2—Ag1^iv^	166.546 (15)	C14—O4—H30	109.5
Au2^iv^—Au2—Ag1^iv^	60.911 (15)	C2—C1—C6	119.7 (14)
Au1—Au2—Ag1^iv^	114.948 (17)	C2—C1—S1	118.4 (10)
Au1^i^—Au2—Ag1^iv^	114.334 (16)	C6—C1—S1	121.9 (11)
Ag2—Au2—Ag1^iv^	69.010 (19)	C1—C2—C3	117.9 (17)
Ag2^i^—Au2—Ag1^iv^	129.17 (2)	C1—C2—H2	121.1
Au1^iv^—Au2—Ag1	166.985 (18)	C3—C2—H2	121.1
Au2^i^—Au2—Ag1	60.820 (17)	C4—C3—C2	123 (2)
Au2^iv^—Au2—Ag1	114.08 (2)	C4—C3—H3	118.3
Au1—Au2—Ag1	60.193 (14)	C2—C3—H3	118.3
Au1^i^—Au2—Ag1	114.789 (17)	C3—C4—C5	118 (2)
Ag2—Au2—Ag1	66.794 (19)	C3—C4—C7	120.0 (18)
Ag2^i^—Au2—Ag1	68.923 (18)	C5—C4—C7	122.3 (19)
Ag1^iv^—Au2—Ag1	129.007 (17)	C4—C5—C6	122 (2)
Au1^iv^—Au2—Ag3^i^	60.189 (15)	C4—C5—H5	119.0
Au2^i^—Au2—Ag3^i^	113.779 (19)	C6—C5—H5	119.0
Au2^iv^—Au2—Ag3^i^	115.005 (19)	C1—C6—C5	119.0 (17)
Au1—Au2—Ag3^i^	166.157 (19)	C1—C6—H6	120.5
Au1^i^—Au2—Ag3^i^	59.858 (15)	C5—C6—H6	120.5
Ag2—Au2—Ag3^i^	131.10 (2)	O1—C7—O2	119 (2)
Ag2^i^—Au2—Ag3^i^	66.867 (18)	O1—C7—C4	121 (2)
Ag1^iv^—Au2—Ag3^i^	68.546 (18)	O2—C7—C4	117.3 (19)
Ag1—Au2—Ag3^i^	129.22 (2)	C13—C8—C9	119.5 (13)
S1—Ag1—S3^ii^	108.36 (8)	C13—C8—S2	117.9 (11)
S1—Ag1—S2	105.62 (8)	C9—C8—S2	121.7 (10)
S3^ii^—Ag1—S2	106.73 (8)	C10—C9—C8	122.3 (16)
S1—Ag1—Au1	140.05 (5)	C10—C9—H9	118.8
S3^ii^—Ag1—Au1	105.21 (6)	C8—C9—H9	118.8
S2—Ag1—Au1	84.43 (5)	C9—C10—C11	116.8 (19)
S1—Ag1—Au2^i^	105.46 (6)	C9—C10—H10	121.6
S3^ii^—Ag1—Au2^i^	82.46 (5)	C11—C10—H10	121.6
S2—Ag1—Au2^i^	142.58 (5)	C12—C11—C10	119 (2)
Au1—Ag1—Au2^i^	58.274 (14)	C12—C11—C14	125.9 (19)
S1—Ag1—Au2	81.31 (5)	C10—C11—C14	114.8 (17)
S3^ii^—Ag1—Au2	140.57 (5)	C11—C12—C13	123 (2)
S2—Ag1—Au2	107.02 (6)	C11—C12—H12	118.4
Au1—Ag1—Au2	58.907 (14)	C13—C12—H12	118.4
Au2^i^—Ag1—Au2	58.271 (14)	C8—C13—C12	117.8 (18)
S1—Ag1—Ag2	100.38 (6)	C8—C13—H13	121.1
S3^ii^—Ag1—Ag2	148.23 (6)	C12—C13—H13	121.1
S2—Ag1—Ag2	51.02 (6)	O3—C14—O4	119 (2)
Au1—Ag1—Ag2	55.883 (16)	O3—C14—C11	121 (2)
Au2^i^—Ag1—Ag2	102.96 (2)	O4—C14—C11	109 (2)
Au2—Ag1—Ag2	56.227 (16)	C16B—C15—C20B	115.4 (19)
S1—Ag1—Ag3^ii^	149.52 (6)	C16A—C15—C20A	122.1 (18)
S3^ii^—Ag1—Ag3^ii^	50.08 (6)	C16A—C15—S3	116.9 (14)
S2—Ag1—Ag3^ii^	101.90 (5)	C16B—C15—S3	125.0 (13)
Au1—Ag1—Ag3^ii^	55.203 (16)	C20B—C15—S3	119.6 (16)
Au2^i^—Ag1—Ag3^ii^	55.866 (15)	C20A—C15—S3	120.7 (13)
Au2—Ag1—Ag3^ii^	102.99 (2)	C15—C16A—C17A	119 (2)
Ag2—Ag1—Ag3^ii^	107.05 (2)	C15—C16A—H16	120.6
S1—Ag1—Ag2^i^	50.32 (6)	C17A—C16A—H16	120.6
S3^ii^—Ag1—Ag2^i^	100.92 (6)	C18—C17A—C16A	122 (3)
S2—Ag1—Ag2^i^	148.59 (6)	C18—C17A—H17	118.8
Au1—Ag1—Ag2^i^	102.58 (2)	C16A—C17A—H17	118.8
Au2^i^—Ag1—Ag2^i^	55.174 (16)	C17B—C16B—C15	126 (2)
Au2—Ag1—Ag2^i^	55.243 (16)	C17B—C16B—H15A	117.1
Ag2—Ag1—Ag2^i^	107.85 (2)	C15—C16B—H15A	117.1
Ag3^ii^—Ag1—Ag2^i^	107.22 (2)	C18—C17B—C16B	120 (2)
S2—Ag2—S1^iv^	129.54 (8)	C18—C17B—H16D	119.9
S2—Ag2—Au1	87.03 (5)	C16B—C17B—H16D	119.9
S1^iv^—Ag2—Au1	141.62 (6)	C17B—C18—C21	123.6 (18)
S2—Ag2—Au2	110.89 (6)	C17A—C18—C21	120.7 (18)
S1^iv^—Ag2—Au2	107.50 (6)	C17B—C18—C19B	120 (2)
Au1—Ag2—Au2	59.513 (14)	C21—C18—C19B	116 (2)
S2—Ag2—Au2^iv^	146.10 (6)	C17A—C18—C19A	117 (2)
S1^iv^—Ag2—Au2^iv^	82.58 (5)	C21—C18—C19A	122.1 (18)
Au1—Ag2—Au2^iv^	59.525 (14)	C20A—C19A—C18	121 (3)
Au2—Ag2—Au2^iv^	58.752 (15)	C20A—C19A—H16A	119.6
S2—Ag2—Ag5	103.59 (6)	C18—C19A—H16A	119.6
S1^iv^—Ag2—Ag5	52.12 (6)	C19A—C20A—C15	118 (2)
Au1—Ag2—Ag5	115.45 (2)	C19A—C20A—H15	120.9
Au2—Ag2—Ag5	144.45 (3)	C15—C20A—H15	120.9
Au2^iv^—Ag2—Ag5	87.64 (2)	C20B—C19B—C18	117 (4)
S2—Ag2—Ag6	51.73 (6)	C20B—C19B—H19A	121.7
S1^iv^—Ag2—Ag6	105.66 (6)	C18—C19B—H19A	121.7
Au1—Ag2—Ag6	87.43 (2)	C15—C20B—C19B	121 (3)
Au2—Ag2—Ag6	145.08 (3)	C15—C20B—H19B	119.3
Au2^iv^—Ag2—Ag6	116.02 (2)	C19B—C20B—H19B	119.3
Ag5—Ag2—Ag6	57.46 (3)	O5A—C21—O6A	123 (2)
S2—Ag2—Ag1	54.15 (5)	O5B—C21—O6B	122 (2)
S1^iv^—Ag2—Ag1	150.39 (6)	O5A—C21—C18	119 (2)
Au1—Ag2—Ag1	56.743 (16)	O5B—C21—C18	118.0 (18)
Au2—Ag2—Ag1	56.979 (16)	O6A—C21—C18	114.6 (19)
Au2^iv^—Ag2—Ag1	104.75 (2)	O6B—C21—C18	120.2 (19)
Ag5—Ag2—Ag1	154.29 (3)	C21—O6A—H6A2	109.5
Ag6—Ag2—Ag1	96.86 (3)	C21—O6B—H5B2	109.5
S2—Ag2—Ag3	101.41 (6)	C27B—C22—C23B	119 (2)
S1^iv^—Ag2—Ag3	99.28 (6)	C27A—C22—C23A	119.2 (19)
Au1—Ag2—Ag3	56.423 (16)	C27A—C22—S4	124.1 (13)
Au2—Ag2—Ag3	104.55 (2)	C27B—C22—S4	120.8 (16)
Au2^iv^—Ag2—Ag3	56.987 (17)	C23B—C22—S4	120.1 (13)
Ag5—Ag2—Ag3	59.06 (2)	C23A—C22—S4	116.5 (14)
Ag6—Ag2—Ag3	59.07 (2)	C24A—C23A—C22	119 (3)
Ag1—Ag2—Ag3	108.82 (2)	C24A—C23A—H23	120.5
S2—Ag2—Ag1^iv^	149.31 (6)	C22—C23A—H23	120.5
S1^iv^—Ag2—Ag1^iv^	51.71 (5)	C23A—C24A—C25	118 (3)
Au1—Ag2—Ag1^iv^	104.28 (2)	C23A—C24A—H24	121.1
Au2—Ag2—Ag1^iv^	55.822 (16)	C25—C24A—H24	121.1
Au2^iv^—Ag2—Ag1^iv^	55.843 (16)	C24B—C23B—C22	119 (2)
Ag5—Ag2—Ag1^iv^	97.15 (3)	C24B—C23B—H23D	120.5
Ag6—Ag2—Ag1^iv^	154.57 (3)	C22—C23B—H23D	120.5
Ag1—Ag2—Ag1^iv^	108.47 (2)	C23B—C24B—C25	118 (3)
Ag3—Ag2—Ag1^iv^	108.64 (2)	C23B—C24B—H24D	121.2
S3—Ag3—S4	130.79 (8)	C25—C24B—H24D	121.2
S3—Ag3—Au1^iii^	108.06 (6)	C26B—C25—C24B	121 (2)
S4—Ag3—Au1^iii^	109.93 (6)	C26B—C25—C28	119 (2)
S3—Ag3—Au1	142.43 (6)	C26A—C25—C28	123.9 (18)
S4—Ag3—Au1	85.00 (5)	C24B—C25—C28	119.3 (18)
Au1^iii^—Ag3—Au1	58.894 (17)	C26A—C25—C24A	120 (2)
S3—Ag3—Au2^iv^	83.88 (5)	C28—C25—C24A	116.3 (18)
S4—Ag3—Au2^iv^	143.50 (6)	C25—C26A—C27A	122 (3)
Au1^iii^—Ag3—Au2^iv^	58.269 (14)	C25—C26A—H26	118.9
Au1—Ag3—Au2^iv^	58.981 (14)	C27A—C26A—H26	118.9
S3—Ag3—Ag5	52.22 (6)	C22—C27A—C26A	122 (2)
S4—Ag3—Ag5	104.65 (6)	C22—C27A—H27	119.1
Au1^iii^—Ag3—Ag5	143.82 (3)	C26A—C27A—H27	119.1
Au1—Ag3—Ag5	115.31 (2)	C25—C26B—C27B	121 (3)
Au2^iv^—Ag3—Ag5	87.42 (2)	C25—C26B—H26D	119.4
S3—Ag3—Ag6	105.57 (6)	C27B—C26B—H26D	119.4
S4—Ag3—Ag6	51.12 (6)	C22—C27B—C26B	121 (3)
Au1^iii^—Ag3—Ag6	144.76 (3)	C22—C27B—H27D	119.3
Au1—Ag3—Ag6	87.75 (2)	C26B—C27B—H27D	119.3
Au2^iv^—Ag3—Ag6	116.23 (2)	O7B—C28—O8B	125 (2)
Ag5—Ag3—Ag6	57.95 (3)	O7A—C28—O8A	124 (2)
S3—Ag3—Ag2	100.26 (6)	O7A—C28—C25	117.9 (18)
S4—Ag3—Ag2	100.28 (6)	O7B—C28—C25	118 (2)
Au1^iii^—Ag3—Ag2	103.22 (2)	O8B—C28—C25	116.0 (19)
Au1—Ag3—Ag2	55.839 (16)	O8A—C28—C25	118.4 (18)
Au2^iv^—Ag3—Ag2	56.138 (16)	C28—O8A—H7AB	109.5
Ag5—Ag3—Ag2	59.50 (2)	C28—O8B—H7BC	109.5
Ag6—Ag3—Ag2	60.13 (2)	C30A—C29—C34A	120 (2)
S3—Ag3—Ag4	149.73 (6)	C34B—C29—C30B	120 (2)
S4—Ag3—Ag4	53.21 (5)	C34B—C29—S5	118.4 (19)
Au1^iii^—Ag3—Ag4	56.773 (18)	C30A—C29—S5	119.6 (18)
Au1—Ag3—Ag4	56.768 (18)	C34A—C29—S5	120.2 (19)
Au2^iv^—Ag3—Ag4	104.28 (2)	C30B—C29—S5	119.7 (18)
Ag5—Ag3—Ag4	154.79 (3)	C29—C30A—C31A	121 (3)
Ag6—Ag3—Ag4	96.90 (3)	C29—C30A—H30D	119.4
Ag2—Ag3—Ag4	108.52 (2)	C31A—C30A—H30D	119.4
S3—Ag3—Ag1^iii^	52.64 (5)	C30A—C31A—C32	113 (3)
S4—Ag3—Ag1^iii^	150.58 (6)	C30A—C31A—H31	123.5
Au1^iii^—Ag3—Ag1^iii^	55.493 (16)	C32—C31A—H31	123.5
Au1—Ag3—Ag1^iii^	103.41 (2)	C31B—C30B—C29	120 (3)
Au2^iv^—Ag3—Ag1^iii^	55.577 (16)	C31B—C30B—H30A	119.9
Ag5—Ag3—Ag1^iii^	97.15 (3)	C29—C30B—H30A	119.9
Ag6—Ag3—Ag1^iii^	155.05 (3)	C30B—C31B—C32	117 (4)
Ag2—Ag3—Ag1^iii^	107.79 (2)	C30B—C31B—H31A	121.5
Ag4—Ag3—Ag1^iii^	107.91 (2)	C32—C31B—H31A	121.5
S4^ii^—Ag4—S4	105.92 (6)	C33A—C32—C35	123 (3)
S4^ii^—Ag4—S4^iii^	105.92 (6)	C33B—C32—C35	119 (2)
S4—Ag4—S4^iii^	105.92 (6)	C33A—C32—C31A	124 (3)
S4^ii^—Ag4—Au1^ii^	83.35 (5)	C35—C32—C31A	113 (2)
S4—Ag4—Au1^ii^	141.29 (6)	C33B—C32—C31B	123 (3)
S4^iii^—Ag4—Au1^ii^	107.34 (6)	C35—C32—C31B	118 (2)
S4^ii^—Ag4—Au1^iii^	141.29 (6)	C32—C33A—C34A	121 (4)
S4—Ag4—Au1^iii^	107.34 (6)	C32—C33A—H32	119.6
S4^iii^—Ag4—Au1^iii^	83.35 (5)	C34A—C33A—H32	119.6
Au1^ii^—Ag4—Au1^iii^	58.23 (2)	C33A—C34A—C29	121 (4)
S4^ii^—Ag4—Au1	107.34 (6)	C33A—C34A—H34	119.6
S4—Ag4—Au1	83.35 (5)	C29—C34A—H34	119.6
S4^iii^—Ag4—Au1	141.29 (6)	C32—C33B—C34B	117 (3)
Au1^ii^—Ag4—Au1	58.23 (2)	C32—C33B—H32A	121.6
Au1^iii^—Ag4—Au1	58.23 (2)	C34B—C33B—H32A	121.6
S4^ii^—Ag4—Ag3	149.88 (6)	C29—C34B—C33B	121 (3)
S4—Ag4—Ag3	51.52 (6)	C29—C34B—H34A	119.6
S4^iii^—Ag4—Ag3	100.24 (5)	C33B—C34B—H34A	119.6
Au1^ii^—Ag4—Ag3	102.99 (3)	O9B—C35—O10B	125 (3)
Au1^iii^—Ag4—Ag3	55.871 (16)	O9A—C35—O10A	121 (3)
Au1—Ag4—Ag3	55.889 (16)	O9B—C35—C32	116 (2)
S4^ii^—Ag4—Ag3^iii^	100.24 (5)	O9A—C35—C32	117 (3)
S4—Ag4—Ag3^iii^	149.88 (6)	O10B—C35—C32	116 (2)
S4^iii^—Ag4—Ag3^iii^	51.53 (6)	O10A—C35—C32	117 (3)
Au1^ii^—Ag4—Ag3^iii^	55.866 (16)	C35—O10A—H10B	109.5
Au1^iii^—Ag4—Ag3^iii^	55.884 (16)	C35—O10B—H10C	109.5
Au1—Ag4—Ag3^iii^	102.99 (3)		

Symmetry codes: (i) *x*−*y*, *x*, −*z*; (ii) −*y*, *x*−*y*, *z*; (iii) −*x*+*y*, −*x*, *z*; (iv) *y*, −*x*+*y*, −*z*.
